# Differences in perinatal morbidity and mortality on the neighbourhood level in Dutch municipalities: a population based cohort study

**DOI:** 10.1186/s12884-015-0628-7

**Published:** 2015-09-02

**Authors:** Amber A. Vos, Semiha Denktaş, Gerard JJM Borsboom, Gouke J. Bonsel, Eric AP Steegers

**Affiliations:** Department of Obstetrics and Gynecology, Division of Obstetrics & Prenatal Medicine, Erasmus MC PO Box 2040, 3000 CA Rotterdam, The Netherlands; Department of Social Sciences, Erasmus University College, Erasmus University Rotterdam, PO Box 1738, 3000 DR Rotterdam, The Netherlands; Department of Public Health, Erasmus University Rotterdam, P.O. Box 2040, 3000 CA Rotterdam, The Netherlands

## Abstract

**Background:**

In a national perinatal health programme, we observed striking heterogeneity in the explanation of the most prominent risks across municipalities. Therefore we explored the separate contribution of several socio-demographic risks on perinatal health inequalities between municipalities and neighbourhoods. The study aims to identify perinatal health inequalities on the neighbourhood level across the selected municipalities, and to objectify the contribution of socio-demographic risk factors on pregnancy outcomes in each municipality by the application of the population attributable risk concept.

**Methods:**

Population based cohort study (2000–2008). Perinatal outcomes of 352,407 single pregnancies from 15 municipalities were analysed. Odds ratios and population attributable risks were calculated. Main outcomes were combined perinatal morbidity (small-for-gestational age, preterm birth, congenital anomalies, and low Apgar score), and perinatal mortality.

**Results:**

Perinatal health inequalities existed on both the municipal and the neighbourhood level. In municipalities, combined perinatal morbidity ranged from 17.3 to 23.6 %, and perinatal mortality ranges from 10.1 to 15.4 ‰. Considerable differences in low socio-economic status between municipalities were apparent, with prevalences ranging from 14.4 to 82.5 %. In seven municipalities, significant differences between neighbourhoods existed for perinatal morbidity (adjusted OR ranging from 1.33 to 2.38) and for perinatal mortality (adjusted OR ranging from 2.06 to 5.59). For some municipalities, socio-demographic risk factors were s a strong predictor for the observed inequalities, but in other municipalities these factors were very weak predictors. If all socio-demographic determinants were set to the most favourable value in a predictive model, combined perinatal morbidity would decrease with 15 to 39 % in these municipalities.

**Conclusions:**

Substantial differences in perinatal morbidity and mortality between municipalities and neighbourhoods exist. Different patterns of inequality suggest differences in etiology. Policy makers and healthcare professionals need to be informed about their local perinatal health profiles in order to introduce antenatal healthcare tailored to the individual and neighbourhood environment.

## Background

It is becoming increasingly clear that health inequalities in western countries are also expressed in adverse perinatal outcomes, such as preterm birth, growth restriction, and perinatal mortality. These adverse perinatal outcomes are especially observed in deprived districts and are often associated with socio-economic and ethnicity related risk factors such as low education, low-income and poor integration into society. Socio-economic status and neighbourhood deprivation are most consistently related to these adverse outcomes [1–4]. Socio-economic status can induce adverse perinatal outcome though multiple pathways, but most importantly through low education and low income levels [5]. However, it is still unclear to what extent the effect of neighbourhood deprivation goes beyond the effect of poor level of socio-economic status at the individual level [6].

Two consecutive reports on perinatal health revealed a relatively unfavourable position of the Netherlands regarding perinatal mortality [7, 8]. Subsequent nationwide cohort studies revealed an equally high impact on perinatal outcomes of non-medical risk factors (e.g. social or lifestyle) compared to medical and obstetrical risk factors [9–11]. In order to gain more insight into these causes and their impact, the concept of ‘Big 4 morbidities’ was introduced [12]. This study showed that four specific defined conditions precede perinatal mortality in 85 % of all cases of perinatal mortality, namely small for gestational age (birth weight < 10th percentile for gestational age) [13], preterm birth (birth < 37 weeks of gestation), congenital disorders, and/or low Apgar score (<7 after 5 min).

Taking this prior knowledge into account, the Healthy Pregnancy 4 All (HP4All) study was initiated to improve perinatal health and to generate effective strategies in disadvantaged areas in the Netherlands [14]. This national study was supported by the Dutch Ministry of Health, Welfare and Sport and combines epidemiologic and health services research to evaluate the effectiveness of two obstetric interventions in preconception care and antenatal healthcare. Municipalities were selected to participate according to socio-demographic data (high risk load) and perinatal outcome data (high adverse outcome prevalence) [14].

Part of the initial fieldwork included consultation with local stakeholders (e.g. caregivers, policy makers) to identify reasons for deprivation. We observed a striking heterogeneity in the explanation of the most prominent risks across municipalities. Unlike the hypothesised homogeneity in deprived areas as known from findings described above, the differences in relative weight of socio-economic and ethnicity related risk factors were much more divergent according to these local stakeholders.

We therefore explored the separate contributions of several socio-demographic risks in neighbourhood perinatal health inequalities in more detail. This study aims (1) to identify perinatal health inequalities on the neighbourhood level across the selected municipalities, and (2) to objectify the contribution of socio-demographic risk factors on pregnancy outcomes in each municipality by the application of the population attributable risk (PAR) concept.

## Methods

### Study population

The selection of the 15 municipalities took place within the Healthy Pregnancy 4 All study. In a thorough preparatory analysis, 50 geographical areas (municipalities) were identified in which adverse perinatal outcomes were high. The list was obtained by combining epidemiological evidence on adverse outcomes from the national perinatal registries of midwives, obstetricians and paediatricians [14, 15, 16]. The 15 municipalities showing the highest perinatal morbidity and mortality rates were selected. All selected municipalities have an above average perinatal mortality rate and have numerous disadvantaged neighbourhoods. Data of pregnant women from these 15 selected Dutch municipalities were analysed in this study. The detailed selection process of these municipalities was described elsewhere [14].

### Study context

The study was conducted in the Dutch antenatal healthcare system. The organisation of the Dutch perinatal care system is unique as, in contrast to most other western countries, midwifery and obstetric care is delivered by primary, secondary and tertiary healthcare providers who function autonomously. At the primary level of care, community midwives provide care to pregnant women with an assumed or estimated low risk for complications during pregnancy and childbirth. Women allocated by the midwife to this low-risk status can opt for a home birth or for an out-patient hospital birth under supervision of their own community midwife. Around 80 % of all pregnant women start their antenatal health care in the primary level of care. If complications threaten to occur during pregnancy or during delivery, women are referred to an obstetrician in a secondary or tertiary hospital [17].

### Data sources

Data from all singleton pregnancies in 15 selected municipalities over the period 2000–2008 were obtained from the Dutch Perinatal Registry (PRN). The PRN committee gave ethical approval for this study (amendment on application 11.36). As the database protects the anonymity of the included pregnant women and data were analysed anonymously, their written consent was not needed.

This registry contains detailed population-based information on pregnancies, deliveries, and neonatal (re)admissions until 28 days postpartum, recorded at the level of the child. Source data were obtained by validated linkage of three independent registries: the midwife registry (routinely collected by 94 % of the midwives), obstetrics registry (collected by 99 % of the obstetricians), and pediatric registry (68 % of the paediatricians including 100 % of the Neonatal Intensive Care Unit (NICU) paediatricians) [15, 16]. Registration of midwifery and obstetric data starts at the first antenatal visit, and complete perinatal data is available from 20 weeks of gestation. The neonatal registry only contains data on hospital admissions of neonates following delivery. Overall, the PRN contains data of > 97 % of all pregnancies in the Netherlands.

For determination of neighbourhood boundaries, we used both 4-digit post codes and municipal neighbourhood boundaries, as established by the national Central Bureau for Statistics (CBS) in 2012 (open access). This last institute is responsible by law for the subdivision of all municipalities in the Netherlands into districts and neighbourhoods amongst others for statistical purposes. This subdivision is based on existing municipal boundaries which occasionally do not coincide with four-digit post code boundaries. As our primary data were post code based, in those instances the post code was assigned to the neighbourhood with the largest share in that particular post code. An exception was made for ‘The Hague’. Historically, this municipality has a different post code classification system resulting in 44 neighbourhoods with many overlapping post codes. We therefore combined several adjacent neighbourhoods and reduced the number to 24 neighbourhoods which allowed for adequate projection of post codes to neighbourhoods. Neighbourhoods containing industrial areas were generally excluded because these areas are non-residential.

### Primary outcomes and determinants

Primary outcomes were perinatal morbidity (Big four) and perinatal mortality. Big four was defined as the presence (single or combined) of small for gestational age (SGA) (birth weight < 10th percentile for gestational age) [13], preterm birth (PTB) (birth < 37 weeks of gestation), congenital disorders (list defined), and/or suboptimal start at birth (Apgar score < 7 after 5 min). Perinatal mortality rate was defined as death in the period from 22 weeks gestational age until 7 days postpartum per 1000 births.

Socio-demographic risk factors included socio-economic status (SES), ethnicity (western, non-western), parity (nulliparous, multiparous) and maternal age. Data on socio-economic status was made available by The Netherlands Institute for Social Research (open access) and provided as status scores on post code level. The SES status scores were composed of four indicators: the average household income per particular post code, the proportion of residents with a low family income, the proportion of poorly educated residents and the proportion of unemployed residents in a particular post code. We divided these status scores into tertiles: below the 20th percentile, between the 20th and 80th percentile, or above the 80th percentile. The post code comprises of socio-economically rather homogeneous small areas with about 25–50 newborn per year. This data was individually linked to the birth record database [19]. Ethnicity was assigned by the caregiver according to the classification of the PRN. The PRN defines ‘ethnicity’ along seven categories in line with the formal guidelines of the CBS: Western Dutch, Western other (including women from other European countries, Australia, and the United States), and non-Western: Mediterranean, (East) Asian, African, South Asian, or other non-Western. The classification of ethnicity recorded in the PRN was made by the health care professional and is typically based on a woman’s appearance, name, and information provided in the context of history taking (at least until January 2015). Note that there is a distinction in the execution of classification between the PRN database and the formal, governmental CBS guidelines where classification was more nationality based on the basis of the information provided by the person (country of birth and parents’ country of birth).

### Statistical analysis

The prevalence of perinatal morbidity and mortality was analysed on the municipal level, and specifically within the selected municipalities.[14] We used data from the years 2000–2008, with the total number of singleton births as denominator. We restricted our data to all singleton pregnancies in the 15 selected municipalities.

Logistic regression was used to study the relation between perinatal morbidity and mortality, and the neighbourhood of residence. The neighbourhood that had the lowest prevalence of adverse perinatal morbidity was chosen as the reference category. These analyses were adjusted for individual factors such as SES, maternal age, parity, ethnicity, and calendar year. In all analyses, municipalities were analysed separately with a significance level set at 0.05. All variables were tested for interaction, and included when statistically significant. Above described statistical analyses were performed using Statistical Package of Social Sciences versions 20.0 for Windows (SPSS Inc, Chicago, IL, USA).

### Population attributable risks

In order to visualise the contribution of socio-demographic risk factors on perinatal morbidity in each municipality, we calculated the population-attributable risk (PAR) percentages. The PAR of a risk factor is the proportion of disease (i.e. pregnancy outcomes) that can be attributed to a specific risk factor only among individuals with the risk factor [20]. In the standard formula, PAR estimations are subject to limitations because the formula is not additive if multiple risk factors interact [11]. Therefore, we followed the staged approach as described by Poeran and colleagues to estimate perinatal morbidity in case selected risk factors were hypothetically absent [11].

The aim of this analysis is to estimate the PAR of socio-demographic risk factors. Therefore, we calculated the PAR for two scenarios. In the first scenario, risk factors were set to ‘the most favourable values’ in terms of outcome whereby all women were ‘assigned’ to the highest SES category (>p80), multiparous, western ethnicity, and 25–29 years old. In the second scenario, risk factors were set to ‘more reasonable values’. Only the women in extreme categories were reassigned: women in the low SES category were assigned to middle SES category (20–p80) and women aged < 18 years or > 35 years were assigned to the reference category ‘25–29 years’. The values in the original dataset remained unchanged.

To estimate perinatal morbidity (Big four), we created a duplicate dataset in which the outcome variables were set to ‘missing values’. We fitted a multivariate logistic regression model on the original dataset to calculate predicted values. The predicted values obtained from the fitted model were used to predict the number of Big four cases for both scenarios, the ‘most favourable values’ and ‘more reasonable values’. For example, the expected number of Big 4 cases in the ‘most favourable scenario’ was estimated by applying the predicted values from the fitted multivariate logistic regression model to the duplicate dataset in which all women were hypothetically reassigned to above listed scenario (e.g. highest SES category).

Finally, the observed Big four cases in the original dataset were compared to the predicted cases of Big four for both duplicate datasets. PARs were estimated as the proportional change of the predicted and observed cases. For this analysis, we used the GLIMMIX procedure in SAS version 9.2 to calculate the predicted values of perinatal morbidity (SAS Institute Inc., Cary, NC).

## Results

A total of 352,407 singleton pregnancies were analysed. The number of pregnancies per neighbourhood ranged from 105 to 16,614 (mean of 2908 pregnancies per neighbourhood).

As a total of 1,584,800 births occurred in the Netherlands during 2000–2008, our study represents 22 % of all births. Considerable differences in prevalences of low SES (prevalences ranging from 14.4 to 82.5 %) and non-Western ethnicity (prevalences ranging from 8.8 to 47.8 %) were apparent across municipalities (Table 1).

**Table 1:** Characteristics of 15 studied Dutch municipalities in 2000–2008

Municipality	Number of residents^a^	Number of births 2000–2008	Number of neighbourhoods	Number of births in low SES (<p20)^b^, N (%)	Number of births in non-western women, N (%)
Almere	193,163	19,302	5	2789 (14.4)	5996 (31.1)
Amsterdam	790,110	90,535	8	58,944 (65.1)	41,897 (46.3)
The Hague	502,055	53,712	24	27,125 (50.5)	22,856 (42.6)
Enschede	158 048	15,312	10	8103 (52.9)	3006 (19.6)
Four villages in the province Groningen	309,244	5850	4	3282 (56.1)	515 (8.8)
Groningen city	193,127	17,372	10	7689 (44.3)	2499 (14.4)
Heerlen	89,016	6864	12	5663 (82.5)	1008 (14.7)
Nijmegen	165 182	15,519	10	7254 (46.7)	2529 (16.3)
Rotterdam	616,260	64,353	15	46,218 (71.8)	30,755 (47.8)
Schiedam	76,244	5715	7	3350 (58.6)	2312 (40.5)
Tilburg	207,580	20,354	10	9548 (46.9)	4528 (22.2)
Utrecht	316,275	37,519	10	1289 (34.4)	10,110 (26.9)
The Netherlands	16,730,348	1,584,800	NA	399,999 (25.2)	257,383 (16.2)

*NA* not applicable, *SES* socio-economic status

^a^In 2012

^b^Defined as status score below the 20th percentile

SGA (ranging from 6.9 to 10.3 %) and PTB (ranging from 5.6 to 7.8 %) determined the largest part in Big four outcomes (17.3–23.6 %) (Table 2). In the years 2000–2008, the average perinatal mortality rate in the Netherlands was 9.5 ‰. In all municipalities, perinatal mortality rates were higher than the national average (10.1–15.4 ‰) (Table 2).

**Table 2:** Perinatal morbidity and mortality rates of the 15 studied Dutch municipalities in 2000–2008

Municipality	SGA (<p10), n (%)	Preterm birth	Congenital anomaly, n (%)	Low apgar score	Perinatal morbidity, n (%)	Perinatal mortality (‰)
Almere	1544 (8.0)	1300 (6.7)	532 (2.8)	222 (1.2)	3788 (19.6)	198 (10.3)
Amsterdam	7397 (8.2)	5677 (6.3)	1763 (1.9)	1307 (1.4)	16707 (18.5)	963 (10.6)
Den Haag	4747 (8.8)	3474 (6.5)	1812 (3.4)	701 (1.3)	11075 (20.6)	590 (11.0)
Enschede	1214 (7.9)	1005 (6.6)	370 (2.4)	226 (1.5)	2290 (19.5)	170 (11.1)
Nijmegen	1274 (8.2)	1048 (6.8)	350 (2.3)	254 (1.6)	2997 (19.3)	191 (12.3)
Groningen city	1226 (7.1)	1073 (6.2)	334 (1.9)	311 (1.8)	3005 (17.3)	175 (10.1)
Four villages in the province Groningen	462 (7.9)	455 (7.8)	126 (2.2)	65 (1.1)	1140 (19.5)	90 (15.4)
Heerlen	709 (10.3)	515 (7.5)	261 (3.8)	90 (1.3)	1618 (23.6)	69 (10.1)
Schiedam	535 (9.4)	448 (7.8)	158 (2.8)	88 (1.5)	1255 (22.0)	75 (13.1)
Rotterdam	5892 (9.2)	4490 (7.0)	1670 (2.6)	973 (1.5)	13668 (21.2)	730 (11.3)
Utrecht	2582 (6.9)	2087 (5.6)	1912 (5.1)	410 (1.1)	6962 (18.6)	415 (11.1)
Tilburg	1850 (9.1)	1384 (6.8)	461 (2.3)	223 (1.1)	4142 (20.3)	212 (10.4)
The Netherlands	111712 (7.0)	97353 (6.1)	44868 (2.8)	18211 (1.1)	281863 (17.8)	15093 (9.5)

Perinatal morbidity is defined as a combined measure of small for gestational age (SGA), preterm birth, congenital anomaly, and / or low Apgar score

### Neighbourhood inequalities

Almost all 15 municipalities showed significant differences between neighbourhoods for both perinatal morbidity and mortality rates. Differences were especially large for perinatal mortality, in which the adjusted odds ratios between the lowest and highest prevalence was 4 to 5 (Table 3). Analyses were adjusted for maternal age, parity, ethnicity, SES, and calendar year effect. The multivariate analysis for the municipality ‘Heerlen’ was not applicable. In this particular municipality, there were only low SES areas. Since neighbourhood and SES are strongly correlated if not identical, it was not possible to make a proper comparison between highest and lowest categories. Interactions between all variables were found to be non-significant. Overall, missing values were less than 1 %. Missing values were set to the most favourable values, e.g. all missing birth weights were set to ‘no SGA’ and included in the analysis (Table 3).

**Table 3:** Difference between neighbourhoods with lowest and highest prevalence of perinatal morbidity and perinatal mortality, expressed as crude and adjusted odds ratios within 15 studied Dutch municipalities

	Perinatal morbidity in neighbourhoods				Perinatal mortality in neighbourhoods			
Municipality	Lowest prevalence n/N, (%)	Highest prevalence n/N, (%)	Crude OR (95 % CI)	Adjusted OR (95 % CI)^a^	Lowest prevalence n/N, (‰)	Highest prevalence n/N, (‰)	Crude OR (95 % CI)	Adjusted OR (95 % CI)^a^
Almere	16/105 (15.2)	2268/11248 (20.2)	1.40 (0.82–2.40)	1.32 (0.77–2.27)	56/6009 (9.5)	123/11248 (10.9)	1.18 (0.86–1.62)	1.11 (0.80–1.54)
Amsterdam	2317/14801 (15.7)	2871/10677 (26.9)	2.00 (1.88–2.13)	1.59 (1.48–1.70)	104/14801 (7.0)	102/9675 (10.5)	3.03 (2.39–3.84)	2.06 (1.57–2.71)
Den Haag	312/2181 (14.3)	693/2624 (26.4)	2.15 (1.85–2.49)	1.59 (1.33–1.90)	6/2181 (2.8)	43/2624 (16.4)	6.14 (2.56–14.74)	5.36 (2.07–13.87)
Enschede	63/483 (13.0)	764/3654 (20.9)	1.76 (1.34–2.32)	1.48 (1.11–1.97)	2/483 (4.1)	15/1401 (10.7)	4.19 (0.95–18.40)	4.78 (1.01–22.59)
Four villages in the province Groningen	210/1144 (18.4)	486/2422 (20.1)	1.12 (0.93–1.34)	1.16 (0.96–1.40)	15/1127 (13.3)	38/2422 (15.7)	1.18 (0.65–2.16)	1.79 (0.91–3.52)
Groningen city	82/737 (11.1)	226/1084 (20.8)	2.10 (1.60–2.76)	1.58 (1.17–2.14)	9/1593 (8.1)	14/1084 (12.9)	2.62 (1.23–5.56)	2.94 (1.32–6.55)
Heerlen	72/396 (18.2)	94/346 (27.2)	1.68 (1.19–2.38)	NA	3/755 (4.0)	7/323 (21.7)	5.56 (1.43–21.61)	5.59 (1.43–21.79)
Nijmegen	90/680 (13.2)	441/1633 (27.0)	2.43 (1.89–3.11)	2.38 (1.84–3.07)	5/923 (5.4)	75/1633 (45.9)	8.84 (3.56–21.93)	3.06 (0.66–14.25)
Rotterdam	779/4721 (16.5)	168/638 (26.3)	1.81 (1.49–2.19)	1.33 (1.17–1.51)	2/522 (3.8)	7/397 (17.6)	4.67 (0.96–22.59)	5.24 (1.08–25.47)
Schiedam	321/1675 (19.2)	353/1390 (25.4)	1.44 (1.21–1.70)	1.14 (0.90–1.44)	9/1000 (9.0)	26/1390 (18.7)	2.10 (0.98–4.50)	1.92 (0.89–4.17)
Tilburg	185/1116 (16.6)	583/2513 (23.2)	1.52 (1.27–1.83)	1.06 (0.84–1.33)	3/791 (3.8)	32/2513 (12.7)	4.32 (1.33–14.0)	4.22 (1.25–14.25)
Utrecht	657/4053 (13.9)	1080/4791 (22.5)	1.80 (1.61–2.00)	1.72 (1.48–1.99)	33/3570 (9.2)	51/4791 (10.6)	1.53 (0.99–2.38)	1.10 (0.67–1.79)

Perinatal morbidity is defined as dichotomous measure, where presence of morbidity means the presence of any of the following either single or combined: small-for-gestational age, preterm birth, congenital anomaly, and/or low Apgar score

^a^model 1. Adjusted for maternal age, parity, ethnicity socio-economic status (SES), and year effect

*OR* odds ratio, *NA* not applicable

### Socio-demographic factors

**Fig. 1:**
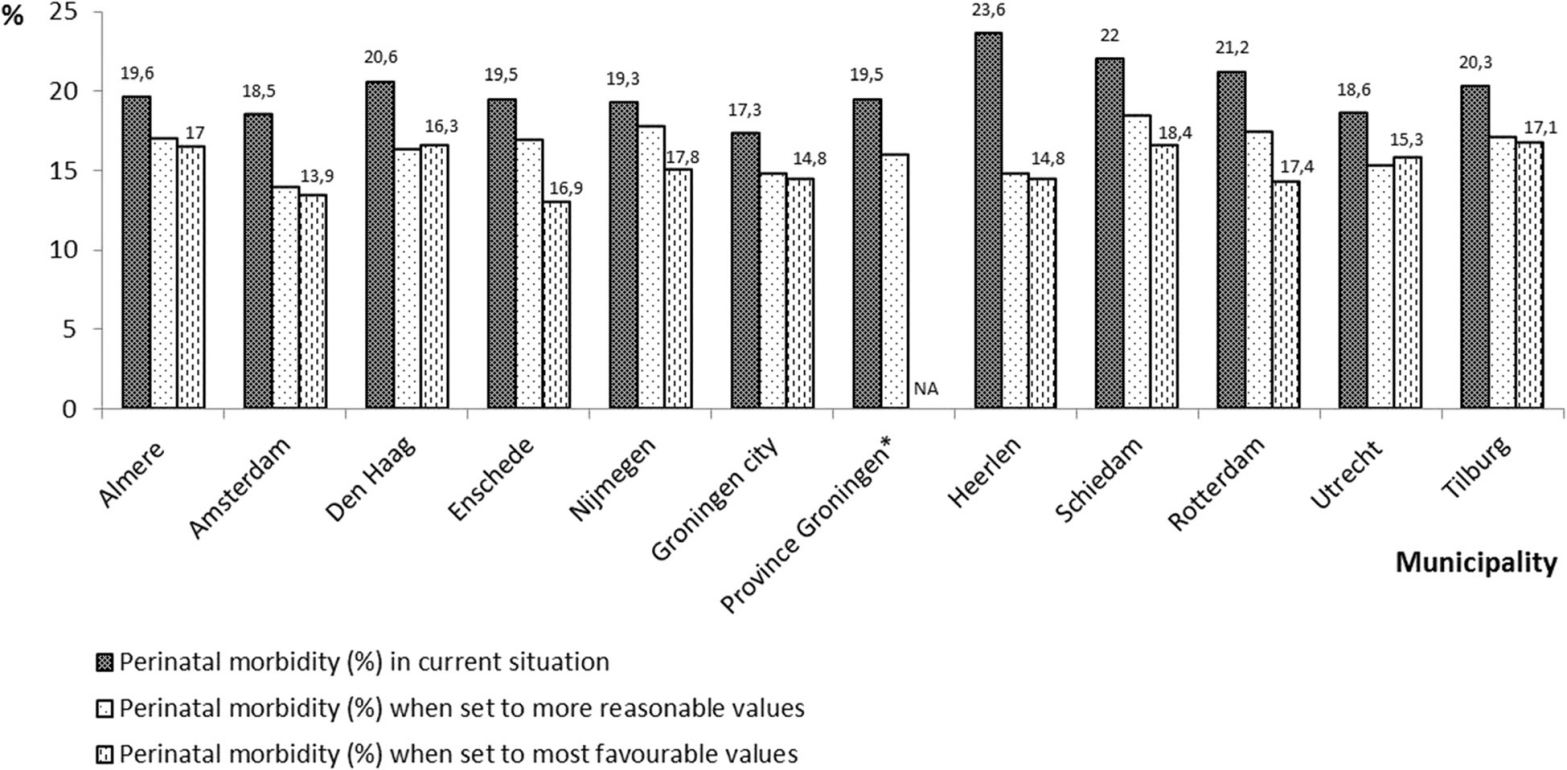
Observed and predicted perinatal morbidity in the 15 selected municipalities. Legend: The selected risk factors were set to ‘the more reasonable values’ and to ‘most favourable values’. In the first scenario, only the women in extreme categories were reassigned: women in the low SES category were assigned to middle SES category (20–p80) and women aged < 18 years or > 35 years were assigned to the reference category ‘25–29 years’. In the second scenario, all women were ‘assigned’ to the most favourable values: highest SES category (>p80), multiparous, western ethnicity, and 25–29 years old. *NA* = not applicable (no cases in highest SES category), *four villages in the province Groningen

In Fig. 1 we displayed the observed Big four outcome of pregnant women in each municipality (from the original dataset), and the predicted Big four outcomes from the duplicate dataset in case of ‘the most favourable’ and ‘most reasonable’ values with the corresponding PARs. In both scenarios, the predicted Big four decreased in all municipalities if socio-demographic risk factors were hypothetically ‘absent’.

With this figure we illustrated that the contribution of socio-demographic risk factors to Big four outcomes differed considerably among municipalities. If-hypothetically-all women would be multiparous, 25–29 years old, of western ethnicity and correspond to the highest SES category (above the 80th percentile), perinatal morbidity would be reduced by 39 % in Rotterdam and 15 % in Utrecht. In other words, the contribution of socio-economic risk factors was nearly one third for some municipalities such as Enschede (33 %), Heerlen (39 %), and Rotterdam (33 %), but appeared to be much lower for others such as the municipalities Almere (16 %) and Utrecht (15 %) (data not shown).

## Discussion

In this study we showed that patterns in perinatal health inequalities differ on both the municipal and neighbourhood level: some municipalities show overall high rates of adverse perinatal outcomes, while others show large differences between neighbourhoods. These neighbourhood differences were particularly pronounced for perinatal mortality. After adjustment for socio-demographic risk factors, such as SES, maternal age, parity, and ethnicity, these differences remained largely present.

These findings are in line with previous studies in which area-level socio-economic variables, such as neighbourhood income or poverty, remained significant after adjustment of individual variables [10, 21, 22]. A previously conducted study in the Netherlands also observed regional differences within the Netherlands, but focused more on care related factors such as travel time [23].

With the use of PARs, we tried to further explore these differences across municipalities by calculating the attribution of socio-demographic risk factors on adverse outcomes. In some municipalities these risk factors are a strong predictor for the observed inequalities by explaining almost a third of the observed differences, while in others their contribution seem less prominent. Behind the general observation that perinatal morbidity and mortality rates are high in these municipalities, different mechanisms are apparently involved. This might be attributed to other explanatory factors, not included in this analysis, such as travel time to a hospital, place of birth, child factors, organisational factors and/or caregiver related factors which are all associated with adverse pregnancy outcomes [11, 24–26]. Two other studies conducted in the Netherlands also calculated PARs by using the same data, but both focused on perinatal mortality and were therefore not entirely comparable [11, 27]. Poeran and colleagues used the same PAR method as we did in our analysis. They estimated the PAR for (the combined effect of) maternal, child and organisational factors. They found a large reduction (over 94 %) in perinatal mortality when all factors were set to the most favourable value [11]. However, this was a nationwide study without focus on area-based differences.

### Strengths and limitations

A major strength of this study was the usage of a validated national perinatal dataset with an almost complete coverage of all pregnancies in the Netherlands over a long period (2000–2008). This dataset includes many variables on both risk and healthcare factors which allowed for detailed analyses. Although the included municipalities had higher rates of perinatal morbidity and mortality than average, they represent one fifth of all pregnancies in the Netherlands. Previous studies were often municipality-based but not nationwide [3]. By including 15 different municipalities, we revealed major differences between areas in a relatively small country and high standards of health care.

This study also has some limitations. Area based measures such as socio-economic status may not correspond to the individual pregnant women, and do not reflect heterogeneity among individuals, healthcare professionals or other characteristic factors within a particular neighbourhood. We dealt with three levels of data (individuals that were clustered in a neighbourhood setting which were in turn nested in cities), so one can consider the use of multilevel type of analysis. Multilevel models address the hierarchical nature of data. Clustering primarily violates the independence of error assumption of most other regression designs [28]. The net effect of multilevel models is a widening of confidence intervals of individual effects, while careful comparison of differences between random slope and random intercept models give an impression on the degree to what level effects actually are present. In the case of the selected 15 countries, we judged that the use of multilevel-modelling was not beneficial. Foremost, this is a comparison of selected cities and neighbourhoods. This aimed a straightforward contrast, rather than a complete neighbourhood study where we felt that multi-level modelling would be more appropriate. We phrased the findings in a non-exaggerating way, which circumvents over optimism with the estimated (individual) intervals.

A limitation was the use of neighbourhood level SES instead of a single individual variable which was unavailable. It has been shown that without adequate control of individual socio-economic factors, neighbourhood effects might act as proxy for unmeasured aspects of unmeasured individual factors [29]. The post code size was small (on average 50 deliveries per year) and post codes were therefore also used as pseudo-individual SES indicator in for example compensation payments to caregivers for the assumed added services in deprivation areas. It has been suggested that the choice of using neighbourhood level variables may be less critical since this captures the unmeasured individual level variation in outcomes and that misspecification of the neighbourhood effect is less likely to occur [30]. However, the invincible use of post code boundaries in our study did not always reflect the actual neighbourhood boundary, which may be a limitation of this study if adjacent neighbourhoods were contrasting [30, 31].

The two greatest advantages of using the PRN database were the large amount of data from pregnancies in the Netherlands that became available over time (over 1 million records) and the high rate of complete cases (more than 97 %). However, by using the PRN database we also faced some limitations. Firstly, the medical registry mainly captures data on specific processes in the healthcare process such as admissions or pregnancy complications. Data on medical, social and pregnancy related risk factors as well as the performance and outcome on prenatal screening are lacking. Using this data for research purposes, other requirements such as the amount and quality of information also become important. One of the disadvantages we faced in our study was the participation of only 70 % of all pediatric wards in the Netherlands (and 100 % of the NICU facilities). This means that partial and selective participation challenges the completeness of short term neonatal outcome. The outcomes reported here, however, are complete as these primarily are recorded by midwife and obstetrician. Secondly, the lack of data of some important maternal risk factors for perinatal morbidity and mortality, such as level of education, smoking during pregnancy, maternal body mass index and folic acid intake was another important limitation of using this database. Smoking is registered in the Perinatal Registry, but this information was not used because of underreporting (prevalence 0.5 %). Thirdly, we faced some limitations in the approach of dichotomous grouping of Western and non-Western women. By dichotomising diverse ethnic groups, socio-demographic characteristics may resemble but groups may differ with respect to patterns of social status, health behavior, biological set up, and consequently birth outcomes. As mentioned in our second limitation, we were unable to study specific risk factor patterns among various ethnic groups, if they should exist. In addition, this approach might result in an oversimplification as this dichotomy might lead to the perception that all non-Western ethnic groups are ‘the same’, reflecting a uniform problem [32]. As we were primarily interested in examining perinatal health inequalities on the neighbourhood level across the selected municipalities, and to objectify the contribution of socio-demographic risk factors on adverse pregnancy outcomes in each municipality by the application of the population attributable risk concept, we opted for this dichotomous classification of Western versus non-Western women. By using two simplified two categories, we tried to evade a potential misclassification due to the allocation of ethnicity on basis of a woman’s appearance in the PRN database. This dichotomous approach was also used in previous studies [32, 33].

### Practical implications and future research

In this study, we observed marked differences in perinatal outcomes across municipalities. We observed different patterns in these disparities: some showed high rates of perinatal morbidity and/or mortality, while others showed large differences between neighbourhoods, or both. Remarkably, socio-demographic risk factors were not always associated with the observed inequalities.

With this study we also emphasise the importance of tailor-made antenatal healthcare, which seems necessary to encounter potential high risk pregnancies. We advise policy makers and health care professionals to develop additional local policy to define their high risk population, e.g. by means of customised preconception care and systemic risk assessment tailored to the individual and social environment of both the woman and the working area of a caregiver. This implicates that more research is necessary to explore etiologic factors associated with perinatal morbidity and mortality on regional level. In 2012 in the Netherlands regional so-called research consortia were constituted to enhance local collaboration which could anticipate to our findings. In addition, more research is necessary to develop specific recruitment strategies to timely reach high risk populations.

## Conclusions

In conclusion, substantial differences in perinatal morbidity and mortality between municipalities and neighbourhoods exist. Socio-demographic risk factors in municipalities are not always a strong predictor for the observed inequalities, implicating that different mechanisms are involved. Our findings suggest that the identification of perinatal morbidity and mortality rates, organisational features of care and etiologic factors on regional level are a valuable first step to customise antenatal healthcare.

### Availability of supporting data

The data set supporting the results of this article is available via Central Bureau of Statistics (http://www.cbs.nl/nl-NL/menu/themas/dossiers/nederland-regionaal/cijfers/incidenteel/maatwerk/wijk-buurtstatistieken/kwb-recent/default.htm.) and via The Netherlands Institute for Social Research (http://www.scp.nl/english/).

## Abbreviations

HP4All: Healthy pregnancy 4 All
NICU: Neonatal intensive care unit
OR: Odds ratio
PAR: Population attributable risk
PRN: Perinatal registry Netherlands
R4U: Rotterdam reproductive risk reduction
PTB: Preterm birth
SES: Socio-economic status
SGA: Small for gestational age
